# On Stomatitis Materna

**Published:** 1853-04

**Authors:** William H. Byford

**Affiliations:** Professor of Theory and Practice of Medicine in Evansville Medical College.


### ARTICLE XXIV.

*On Stomatitis Materna.*—By William H. Byford, M. D. Professor of Theory and Practice of Medicine in Evansville Medical College.

As its name implies, this is a disease peculiar to those who are, or who are about to be, mothers, and is attended with painful inflammation of some portion of the lining membrane of the mouth. Although inflammation of the mouth is a symptom considered necessary to the full development of the disease, it must be regarded only as a symptom attending a general disorder of the whole system, or at least of some one of its constituents, perhaps the blood, which by its own peculiar modification implicates the solid parts in an action which they would not otherwise take upon themselves. This view of its pathological seat, it is believed, is the only one which will enable us satisfactorily to account for many of the phenomena presented, both in respect to the time of occurrence. and the particular solid tissues mostly affected. What this modification of the condition of the blood may be, we can only conjecture; as, in the present state of science, the investigations, which have extended only to the physical and chemical qualities of this fluid, do not afford the means of ascertaining with any exactitude many of the most important changes which occur in it.

What are its vital conditions under varied circumstances, or indeed in any case, perhaps, is entirely beyond the reach of our philosophy, and it may be, will ever elude the imperfect means of search attainable by the ingenuity of man. A deviation in the vital, physical, or chemical properties of the blood from the healthy standard, must produce effects commensurate to the importance of each, and the amount of departure from this normal state.

That there are many reasons for believing this disease to be the result of some kind of modification of the conditions of the blood, I think will be admitted by most who have thought much upon the subject. The nature of the alteration we do not now know. Could we see all the vital and chemical changes produced in the blood—for changes there must be—by the efforts of the mother's system to sustain the child *in utero*, and the necessary mutation after the process of gestation is completed, compared with its condition in health and during lactation, we might receive some light upon the subject. I apprehend that we should be taught by such instructive lessons that the character, in some respects, of change in the blood itself, would be continuously the same from the commencement of gestation to the termination of lactation; and that the effect upon the mother's energies would correspond with the quantity of the peculiar change growing out of the increasing wants of the being dependent upon her for support. And as anemia is one of the results of protracted lactation, this tendency may begin with the commencement of pregnancy. Patients not very competent to the task of childbearing and its consequences, however, often become enfeebled and thin in gestation, and continue to become more so until they succumb, or are relieved from the discharge of these duties. The same nourishing principle called for to sustain the child *in utero* is still demanded to nourish it at the breast; and it may be a secretion in the first place, similar in character (possibly identical) to what it is in the last. This demand increases as the child grows in size and vigor, and must consist of the proximate elements of nutrition so nearly vitalized as to require none in the first instance, and in the second but feeble powers of digestion to render them subservient to their appropriate uses. If the pathological condition of the system is that of anemia resulting from pregnancy and lactation, there must be some peculiarity about it, judging from its effects, differing from anemia arising from other causes. And this may be the peculiarity, viz. a greater paucity, comparatively, perhaps, than in any other case of anemia of the material elements expended in the process of nutrition. And this may be the case without any of the ordinary signs of anemia. From what has been said above, it may be inferred that pregnancy, as well as lactation, is regarded by me as one of the causes of stomatitis materna. Several cases have occurred in my practice in women who were pregnant with their first child, which continued throughout the remaining time of gestation and during lactation. I remarked that all the patients in whom these cases occurred. were very young, of scrofulous diathesis, weakly, and labored under most of the symptoms of anemia resulting from other exhausting influences, such as pallor, languor, shrunken veins, &c. I think that without other influences, pregnancy and lactation are not sufficient; and hence I believe that we must look for extrinsic causes. And these, probably, are both endemic and epidemic. By the former, I mean such morbific agents as are operating in the immediate locality of the patient; for, so far as I can learn, it is not of very general prevalence. By the epidemic influence, I mean the extensive change which has taken place in the general cast of the diseases of the West, especially along the course of the large rivers, from the ordinary endemic bilious fevers, and other miasmatic diseases, to the typhoid or continuous type, attended for the most part with affections of the mucous membranes, particularly of the alimentary canal. This typhoid diathesis is so remarkably developed in many parts of this extensive tract of country, that it has usurped the place of the former diseases, or moulded them into a different type. The western physician of many years experience, can remember that in many extensive sections of country nothing was known from observation, until within a few years past, of the typhoid or continued fever. Taking these things into consideration, I think it may be inferred that a wide spread epidemic influence has been for some time exerting its force upon the inhabitants of this portion of the country, producing a condition of the system in which there is strong proclivity to disease of the mucous surface; and operating upon the maternal system, its effects are the tendency to this disease; just as in the particular seasons, and under certain circumstances, there is a tendency to puerperal fever. There are three different varieties of this disease. The first includes the most simple variety, so far as local symptoms are concerned. It is characterized by superficial and often diffused inflammation of the mucous membrane of the mouth, which may be confined to a small part, as the lips, or end of the tongue; or it may spread throughout the whole cavity of the mouth and fauces. The parts, upon examination, are found of a scarlet red color, and dry; but as a general thing not much, if at all swollen. This appearance may be of transient duration, lasting, probably, only a few hours; more generally, however, for several days, when in a great many instances it completely subsides, leaving the patient to all appearances quite well, with the exception of a little debility. In some rare cases the subsidence is not so complete, and amounts only to a very considerable remission of the soreness and distress. After an uncertain length of time—in slight cases, longer, and shorter in severe ones—the inflammation returns, and runs a similar course to the former paroxysm. The paroxysms usually commence suddenly, with a sense of burning or scalding in some part of the mouth—oftener perhaps on the end of the tongue than elsewhere—which rapidly spreads, involving the parts continuously, until the whole mouth feels like it had been scalded, and the acts of mastication and deglutition are intolerably painful. The subsidence of the pain and suffering is as sudden and gratifying as it was unexpectedly afflicting.

The second variety seems to engraft itself, as it were, upon the diffuse and superficial inflammation of the first; for, in addition to the appearances above described, a crop of vesicles are scattered over the whole or a part of the inflamed surface. These are often so clear and transparent that without attention they may be easily overlooked; sometimes, again, they have an aphthous appearance, and are quite obvious. The duration of this eruption is about eight or ten days; but it often lasts much longer, and then consists of successive crops. The symptoms, though they subside sometimes as completely as in the former variety, the respite from suffering, is commonly shorter and less complete. In the progress of a case, it is not unusual for the appearance described as the two varieties to alternate with each other.

In the third variety, the whole force of the paroxysm is concentrated upon a small part of the surface, always, in my experience, upon the tongue. Either upon its side or inferior surface I have seen it begin by a fissure gradually leaving an ulcer, from a hardened tubercle, from the bursting of a vesicle, or simply from an inflamed point. However it may commence, a rapid ulceration destroys the substance of the tongue, until a ragged notch has half completed its amputation. Suddenly it ceases, the cavity granulates, fills up, and heals, but the organ is left distorted. The patient flatters herself, upon the cessation of each paroxysm, that some newly-applied remedy is an all-sufficient sanative against the ills which she knows, by past experience, is in reserve for her. But, unfortunately, with a returning paroxysm, she finds her suffering unmitigated. Notwithstanding this fearful ulcerative process, this variety is less dangerous, and affects the constitution more mildly than either of the other varieties. A very important consideration in connection with the local manifestation of the disease is, that the two first varieties are migratory, travelling from the mouth along the surface of the mucous membrane to all the neighboring cavities, down through the pharynx and œsophagus to the stomach; and thence through the whole extent of the alimentary canal, frequently finding permanent lodgment in some section of this extensive tube, and destroying the patient by originating chronic gastritis, duodenitis, ileitis, &c., or passing through the larynx, trachea, and into the bronchia. And if it does not, by establishing inflammation in some portion of these tubes, exhaust the patient, it may awaken into existence the more fatal affections of the substance of the lungs. It has also followed the nasal passages into the different cavities of the skull, or maxillary antrum, and there induced permanent inflammation. At other times it travels through the Eustachian tube to the tympanum, and thence to the mastoid cells. And I have seen one case where permanent deafness of one ear and exfoliation of bone from the mastoid process occurred. The most common course for it to take, is into the alimentary canal and lungs. It is very prone to fasten fatal disease upon the lungs when it is allowed to run on for any considerable length of time.

The date of the commencement of the above local symptoms is various. Hitherto, I believe, it has been considered that they date from some time during the term of lactation, especially where the subject is young and with the first child, but that they might reappear during subsequent pregnancies; and that they never are present in pregnancies unless the patient has been subject to the disease during some previous term of lactation. I am not certain that this is the general rule. But it is unquestionably the fact that a woman will be more likely to experience such trouble after having once labored under the malady.

Accompanying the above array of symptoms are those of a general character. Perhaps, of all others, disorder of the digestive organs is the most prominent, as well as first in importance. Difficulty of deglutition, indigestion, and diarrhea, form a part of these. All of these symptoms, like the local, are more or less paroxysmal; the diarrhea particularly. For some time it will harass the patient, exhaust the resources of her system, and prevent rest, and then disappear; and allow her to recruit strength, to be prostrated again by its return. Indigestion is, probably, more or less constantly present. Difficulty of mastication and deglutition vary of course with the local symptoms. Emaciation is also often considerable, and generally keeps pace with the digestive disorders. Many other general symptoms might be enumerated, but as they are not peculiar or so important, they will not be noticed. It is sufficient to be aware that such extension of the inflammation does occur, &c.

The first step in the treatment of any disease should be the removal of the cause, when practicable. In cases where more than one cause contributes to the production of disease, the removal of one of them may so far interrupt the chain of impressions as to accomplish a cure. Occasionally, this is found to be the case in the disease under consideration. The patient, by a change of her residence for the balance of the time of lactation, may get quite well. This, however, is not generally the case. And in all instances where the objections are not too weighty, the child should be removed or transferred to another nurse. I have seen so many cases of unfortunate terminations, and regard the condition of my patient so uncertain while laboring under this ubiquitous inflammation, if I may be allowed the term, in which, without any warning, the precincts of some vital organ is involved, and it becomes the seat of destructive organic alteration, that I deem it a matter of great importance to take *immediately* the most effectual course within my knowledge to place the patient under the most favorable circumstances. After an experience of nine years duration, I cannot feel quiet while my patient, if at all seriously affected, continues to nurse her child. I am thoroughly convinced that, in many instances which I have known to be followed by fatal secondary diseases, had the connection between the sore mouth and these been properly appreciated, and the causes of it understood, the mother's life would not have been sacrificed in a useless attempt at nursing; and I am well assured that, with the best management in grave cases, there is much more likelihood of the patient becoming worse than better while she nurses her child. I will also say that, in some cases, weaning will not of itself cure, although this is the general rule; and so soon as any of the serious complications are lighted up, little is to expected for them from this measure. Should the case not yield to weaning, and change of residence, or should they be impracticable, or from any reason not be adopted, all that can be expected from medical treatment is that it may *palliate* the symptoms, and perhaps prevent the disease from advancing. The most obvious and urgent indication for treatment will be found in the emaciation and debility. We must meet them as best we can with tonics and proper diet. Animal food, in as liberal quantities as the enfeebled digestive apparatus will allow, must be given. Milk, eggs, and mutton I have found to agree best. First upon the list of medicines may be placed cod-liver oil, which I have been in the habit of recommending for the last three years. It must be persevered in during the whole term of lactation, and as long after as any traces of the disease exist. The dose should be regulated by the capacity of the stomach, as much being given as can be retained. This remedy has the advantage of any other alterative and tonic in its soothing effects upon the irritated bowels when this difficulty exists. This is a circumstance of the utmost importance, as we are often interrupted in the use of remedies by diarrhea.

Where there is no diarrhea, or between the paroxysms of it, the most useful tonic next to the oil is the carb. of iron, prepared and used according to the following formula: ℞. Carb. potass. sulph. ferri, āā ʒ iss; gum. acacia mucil. ℥ iv. Pulverize the potass, and dissolve in the mucilage; then pulverize and add the sulph. of iron; mix well in an earthen mortar. Dose, half an ounce three times a day, gradually increasing to as much as the stomach will bear. For persons very much reduced, of lax habit, brandy or wine taken during meals will sometimes do good. Very often they both disagree, when we may substitute ale, porter, or beer, among the malt liquors. Vegetable tonics and aromatics are useful in certain cases, where the iron disagrees with the stomach.

The diarrhea, so exhausting to the patient and perplexing to the physician, should claim our attention also. Indeed, while this symptom exists, our efforts to restore the lost energies of the system and natural condition of the blood, from our inability to introduce effective tonics, will be futile.

Entire quietude, while this is in active existence, in the horizontal posture, should be strictly enjoined. Morphia, combined with acetate of lead, will often control it very completely. We may replace the latter remedy by sulphate of copper or nitrate of silver. These latter, when given with solid opium, often answer an excellent purpose in quieting the irritation of the bowels, and acting also as tonics to the system. Astringent injections and suppositories may be used. Indeed, in some instances, all our resources will be vainly tried to relieve this symptom. A great variety of local remedies has been put in requisition, and all have enjoyed more or less praise. I have used with benefit a solution of sulphate of copper, of different degrees of strength, the vinous tincture of golden seal, a weak solution of nitric acid, sulphate of alum, borax, and sulphate of zinc; some of them agreeing in one case, and some with another. Perhaps among them all, a solution of sulphate of copper did good most frequently. The only way I have ever been able with certainty to adapt the local remedies to different cases was by trying them.

In conclusion, I wish to embody, under a few distinct heads, the result of my experience and reflection upon the subject. 1st. It is a disease of pregnancy and lactation, more frequently appearing while the patient is in the discharge of the last-named function, especially should it be the first child. Certainly, however, often making its appearance during pregnancy with the first child. 2d. The condition of blood probably gives origin to the local manifestation of the disease. This condition of the blood may arise from the abstraction, to too large an extent compatible with the health of the mother's system, of such principle or principles as may be necessary for the support of the child, either through the placenta or the mammæ, depraved digestion and assimilation, and other depressing circumstances connected with certain epidemic visitations and endemic tendencies. 3d. The local symptoms are irregularly paroxysmal. 4th. It makes its appearance in three distinct forms, viz. erythmatous, vesicular, and ulcerative inflammation of some part of the mouth. The former two generally covering the whole internal surface of the mouth; the latter usually confined to the tongue. 5th. The first two varieties are migratory, spreading, in different cases, to all the mucous membrane continuous with the cavity of the mouth; such as that lining the air-passages, the lungs, the digestive surface, the cells and cavities of the cranium, maxillary nasal sinuses, &c.; thus producing consequences varying in gravity and other characteristics with the constitutional tendency of the patient and the amount and seat of the inflammation set up on these surfaces. 6th. The prognosis is doubtful even in cases that seem favorable, from the complications that may arise by its spreading character. 7th. That in cases of gravity, medicine will avail but little without a change of residence or nursing, or both. 8th. Cod-liver oil and tonics, especially the ferruginous, and nutritious diet, are the main hope of success in the simple form. The complicated, of course, will demand remedies suited to the circumstance at the time, and calculated for the same diseases when produced from other causes. 9th. Local remedies are merely palliative.

[*Am. Jour. Med. Sci.*

